# Human-alignment influences the utility of AI-assisted decision making

**DOI:** 10.1038/s41598-025-12205-1

**Published:** 2025-08-09

**Authors:** Nina L. Corvelo Benz, Manuel Gomez Rodriguez

**Affiliations:** 1 Max Planck Institute for Software Systems, Kaiserslautern, 67663 Germany; 2 Department of Biosystems Science and Engineering, ETH Zurich, Basel, 4056 Switzerland

**Keywords:** Computer science, Human behaviour

## Abstract

Whenever an AI model is used to predict a relevant (binary) outcome in AI-assisted decision making, it is widely agreed that, together with each prediction, the model should provide an AI confidence value. However, it has been unclear why decision makers have often difficulties to develop a good sense on when to trust a prediction using AI confidence values. Very recently, Corvelo Benz and Gomez Rodriguez have argued that, for rational decision makers, the utility of AI-assisted decision making is inherently bounded by the degree of alignment between the AI confidence values and the decision maker’s confidence on their own predictions. In this work, we empirically investigate to what extent the degree of alignment actually influences the utility of AI-assisted decision making. To this end, we design and run a large-scale human subject study ($n = 703$) where participants solve a simple decision making task—an online card game—assisted by an AI model with a steerable degree of alignment. Our results show a positive association between the degree of alignment and the utility of AI-assisted decision making. In addition, our results also show that post-processing the AI confidence values to achieve multicalibration with respect to the participants’ confidence on their own predictions increases both the degree of alignment and the utility of AI-assisted decision making.

## Introduction

State-of-the-art AI models have matched, or even surpassed, human experts at predicting relevant outcomes for decision making in a variety of high-stakes domains such as medicine, education and science ^1–3^. Consequently, the conventional wisdom is that human experts using these AI models should make *better* decisions than those not using them. However, multiple lines of empirical evidence suggest that this is not generally true ^4–7^.

In the canonical case of binary outcomes and binary decisions, Corvelo Benz and Gomez Rodriguez ^8^ have recently argued that the way in which AI models quantify and communicate confidence in their predictions may be one of the reasons AI-assisted decision making falls short. Their key argument is that, if an AI model uses calibrated estimates of the probability that the predicted outcome matches the true outcome as AI confidence values, as commonly done ^9–14^ , a rational human expert who places more (less) trust on predictions with higher (lower) AI confidence may never make optimal decisions. Further, they show that the optimality gap is proportional to the maximum alignment error (MAE) between the AI confidence and the human expert’s confidence, *i.e.*,

1 $$\begin{aligned} \text {MAE} = \max _{h \le h', a \le a'} P(Y = 1 \,\mid \,A=a, H=h) - P(Y = 1 \,\mid \,A=a', H=h'), \end{aligned}$$

where *A* and *H* are the AI and the expert’s confidence values in the outcome $Y=1$, respectively.

While the above theoretical results are illuminating, they do not elucidate to what extent there is an association between the degree of alignment between the AI confidence and the human expert’s confidence and the utility of AI-assisted decision making—they just show that the maximum utility we can expect from AI-assisted decision making is inherently bounded by the degree of alignment. In this work, we aim to fill this gap by designing and running a large-scale human subject study where participants solve a simple decision making task—a card game—assisted by an AI model with a steerable degree of alignment.

**Fig. 1:**
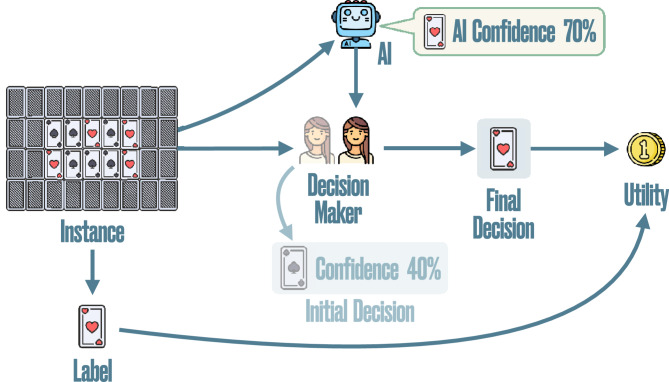
Our simple AI-assisted decision making task.

Our AI-assisted card game does not require prior knowledge besides a very basic understanding of probabilities, participants just need to guess the color of a randomly picked card from a pile of red and black cards that is partially observed assisted by an AI model, as shown in Fig. 1. To control the degree of alignment between the AI confidence and the participants’ confidence, our game biases the proportion of red and black cards shown to the participants according to the AI confidence value.

Participants in our human subject study are randomly assigned to one of four different groups ($n\approx 100$ participants per group). In the first three groups, denoted by the symbols ,  and , participants are assisted by the same AI model, which is perfectly calibrated; however, the degree of alignment varies across groups. In the fourth group, denoted by symbol , participants are assisted by an AI model whose predictions are post-processed to increase their degree of alignment via multicalibration using additional (held-out) data ($n=302$ participants) from the group with the lowest degree of alignment within the first three groups. Participants do not know how the AI assistant generates its predictions nor which group condition they are assigned to (single-blinded). We evaluate the impact of the degree of alignment on the utility of AI-assisted decision making by running multiple Bayesian A/B tests for two categories of games characterized by participants’ initial performance and for pairs of groups , , , and . We find decisive evidence that the utility is greater in groups with higher degree of alignment compared to the group with the lowest degree of alignment for games where participants initially perform badly (Evidence Ratios $>100$ with an estimated difference in utility of greater or equal to 0.15 between groups compared). Moreover, we also find evidence that increasing the degree of alignment via multicalibration increases the utility achieved by participants in the fourth group compared to the reference group before multicalibration for all games (Evidence Ratios of 2.59 and $>100$ with an estimated difference in utility of 0.02 between the two groups for each category of game).

## AI-assisted game design

The game consists of different rounds $i\in \{1,\dots , 24\}$. In each round, the game shows the participant 21 cards out of a game pile of 65 cards and picks one card at random from the pile. Based on the cards shown, it asks the participant to guess the color of the card picked and give their (initial) confidence that the card picked is red. Then, it shows the participant the AI confidence that the card picked is red and allows them to revise their guess and confidence. Finally, the participant wins a point if their final guess is correct and otherwise they lose a point. To incentivize participants, we give a bonus compensation of $\pounds 0.12$ at the end of the game for each point earned.

### A perfectly calibrated AI, by design

Participants in the first three groups are shown 24 different types of game piles *S*, a different type per round in randomized order. Each type of game pile $(r, a) \in S$ is defined by the fraction *r* of red cards in the game pile and the AI confidence *a*. The AI confidence *a* takes values from the set $\mathcal {A}= \{1/13, 2/13 \dots , 12/13\}$ and the fraction *r* of red cards is given by

$$\begin{aligned} r \in \{ a-\text {var}_a, a+\text {var}_a\}, \end{aligned}$$

where $\text {var}_a$ is chosen such that, in Eq. 2, $r\cdot 65$ is an integral number of cards for all *r*. To ease interpretability, participants are shown re-scaled AI confidence values rounded to the nearest integer between 0 and 100. As *r* determines the probability that the game picks a red card from the game pile and each participant is shown each type of game pile once, the AI confidence is perfectly calibrated, *i.e.*, for all $a \in \mathcal {A}$, it holds that

$$\begin{aligned} P(C=\texttt {red} \,\mid \,A=a) = a\,. \end{aligned}$$

We also validated empirically that the rounded AI confidence values indeed obtain a low calibration error as well (see Supplementary Table S1 online).

Further, since one can argue that the AI model has essentially information about only 13 cards whereas participants have information about 21 cards, the latter have more information about each pile than the former. However, participants in all groups are oblivious to this—they are said that, while the AI sees all cards in the pile, the AI confidence may not be fully accurate due to unknown errors (see Supplementary Fig. S13 online). We chose this exposition as to simplify instructions and conceal information about the group assignment.

### Steering alignment by biasing the proportion of red and black cards shown to the participants

In each round, the 21 cards of the game pile shown to the participants in the first three groups are sampled from a Wallenius’ noncentral hypergeometric distribution with an odds ratio $\omega _g(r,a )$ defined by the group *g* participants are assigned to.

The odds ratio $\omega _g(r,a )$ biases the likelihood of the color ratio in the cards shown to the participants—if $\omega _g(r,a )$ is greater (smaller) than 1, it is more likely to sample a red (black) card than a black (red). More formally, the fraction *z* of red cards shown to the participant is given by

2 $$\begin{aligned} z \sim \text {wnchypg}\left( 21, 65\cdot r , 65\cdot (1-r ), \omega _g(r ,a )\right) . \end{aligned}$$

and, for each group *g*, the odds ratio $\omega _g(r,a )$ biases the generation as follows:

- **Group** : Bias the fraction of reds shown towards the AI confidence—the odds of sampling reds (blacks) is greater than the odds of sampling blacks (reds) when the true probability *r* is lower (greater) than the AI confidence *a*. We set
  $$\omega _g(r ,a )= {\left\{ \begin{array}{ll} 1/4 \quad \text {if } r > a , \\ 4 \quad \text {if } r < a . \end{array}\right. }$$
- **Group** : Bias the fraction of reds shown away from the AI confidence—the odds of sampling reds (blacks) is greater than the odds of sampling blacks (reds) when the true probability *r* is greater (lower) than the AI confidence *a*. We set
  $$\omega _g(r ,a )= {\left\{ \begin{array}{ll} 4 \quad \text {if } r > a , \\ 1/4 \quad \text {if } r < a . \end{array}\right. }$$
- **Group** : No bias. We set $\omega _g(r,a )= 1$.

**Fig. 2:**
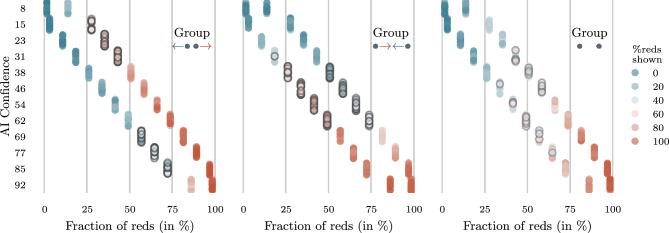
AI confidence *a* and fraction of red cards shown *z* against the fraction of red cards *r* per game played by participants in each group. Games in which the participant are most likely mislead since the majority of cards shown have the opposite of the overall majority color are highlighted in gray.

The resulting empirical distribution of red cards shown to the participants across game piles is shown in Fig. 2. In Group , we bias the fraction of reds shown towards the AI confidence, when conditioning on the AI confidence. Therefore, we expect that participants have lower (higher) confidence in guessing red for games where the true probability of picking a red card in the game pile is higher (lower) than the AI confidence leading to lower degree of alignment (see Eq. 1). However, in Group  and , we expect the opposite to happen and, thus, the AI to have a higher degree of alignment with participants. Here, note that, the quantity and type of games where participants may perform poorly are different across groups. In particular, the unbiased Group  has fewer game instances compared to the groups  and  in which the participant may be misled by the color ratio of the cards shown.

### Increasing alignment via multicalibration

Participants in the Group  are shown the same type of game piles with the same fraction *z* of red cards shown as participants in the Group . However, the AI confidence is post-processed by the uniform mass histogram binning algorithm introduced by Corvelo Benz and Gomez Rodriguez ^8^ for the purpose of multicalibration. We run the algorithm with additional (held-out) calibration data from the Group  for a different set of games from the same distribution. Refer to the Materials and Methods section and Supplementary Fig. S3 online for more information regarding the post-processing algorithm and the calibration data.

## Results

**Fig. 3:**
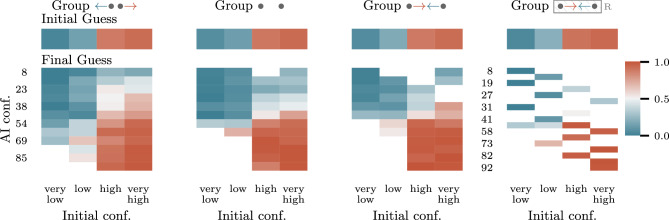
Heatmap of decision probabilities of guessing red stratified by participants’ initial confidence and AI confidence shown, and averaged over participants. The initial confidence recorded is discretized into four bins—very low, low, high, very high—denoting the confidence of the participants that the color of the picked card will be red. Bins with 10 or less data points are not displayed.

We first quantify the degree of alignment between the AI confidence and the human expert’s confidence for each group by measuring the empirical maximum alignment error (MAE, Eq. 1) and expected alignment error (EAE, Eq. 3). Table 1 summarizes the results. We observe the lowest alignment error in Group , closely followed by Group , and the highest alignment error in Group , where we observe more than twice higher MAE and a 20 times higher EAE than in Group . Further, in Group , we observe that the multicalibration algorithm helps to reduce the EAE by a third and the MAE by almost half in comparison to Group . In addition, in Supplementary Fig. S4 online, we also observe that, within each group, the initial confidence of participants is normally distributed around the percentage *z* of reds shown. Based on these findings, we conclude that, as expected, our AI-assisted game design has successfully steered the degree of alignment in each group.

Further, we verify that, on average, participants behave rationally in all groups, which is one of the motivating assumptions underpinning our work. More specifically, in Fig. 3, we observe that the average probability that a participant’s initial guess is red depends monotonically on the participant’s own confidence that the card chosen is red and the probability that a participant’s final guess is red depends monotonically on both the participant’s own confidence and the AI confidence that the card chosen is red. As an immediate consequence, the maximum utility of AI-assisted decision making we can hope for is bounded by the degree of alignment in each group, although with minimal differences (see Supplementary Fig. S1 to S3 online and Supplementary Information online under Additional Experimental Results).

**Table 1:** Expected alignment error (EAE) and maximum alignment error (MAE) for each group.

Group	EAE	MAE
	0.00065	0.1
	0.0003	0.06
	0.00693	0.2
	0.00236	0.12

Next, we contrast the utility achieved by participants across groups controlling for the different quantities and types of game piles across groups. To this end, we compare the initial guess *d* and the final guess $d'$ made by each participant in each game against the optimal guess $\pi ^* = \pi ^*(r)$, where $\pi ^*(r )$ is red if and only if the fraction of red cards $r >0.5$ (there are no games with $r =0.5$). More formally, we focus on the conditional matching rates

$$\begin{aligned} \theta _1 = \mathbb {E}[Q' \,\mid \,Q =1] \quad \text {and} \quad \theta _0 = \mathbb {E}[Q' \,\mid \,Q =0] \end{aligned}$$

where the expectation is over games and participants and

$$ Q := \mathbbm {1}[\pi ^*(r ) = d ] \quad \text {and} \quad Q' := \mathbbm {1}[\pi ^*(r ) = d' ]. $$

The conditional matching rates measure how close participants are on average to the optimal decision for each game after observing the AI confidence given that they were initially optimal or not optimal. Comparing conditional matching rates instead of other utility measures has two main benefits: For one, this measure is more comparable between groups, because, for a given type of game pile, the optimal guess is the same across groups for each participant since $\pi ^*$ depends only on *r*. Furthermore, because we compare rates conditional on the initial performance *Q* of the participants, we take into account the effect of different amounts of games in each group where participants are misled.

To evaluate the impact of the degree of alignment on the participants’ conditional matching rates in each group, we conduct multiple Bayesian A/B tests: We first test if the conditional matching rates $\theta _0$ and $\theta _1$ are greater for groups  and  with higher degree of alignment than for Group  with lower degree of alignment. We then test if the conditional matching rates $\theta _0$ and $\theta _1$ are greater after post-processing for Group  than for Group . To this end, we fit two Bayesian binomial (logit) mixed effects models for the (unconditional) matching rate $\theta = \mathbb {E}[Q']$ with an interaction coefficient between the group condition *g* and *Q*, and a random intercept for each participant, *i.e.*,

$$\log \left( \frac{\theta }{1-\theta }\right) \sim 0 + g * Q + (1 \,\mid \,\text {participant}).$$

We opt for no fixed global intercept in order to not have a fixed reference group in the model. However, fitting the model with a fixed global intercept returns equivalent results. Note that, the conditional matching rates $\theta _0$ and $\theta _1$ can be recovered from the model by setting $Q=0$ and $Q=1$, respectively.

**Fig. 4:**
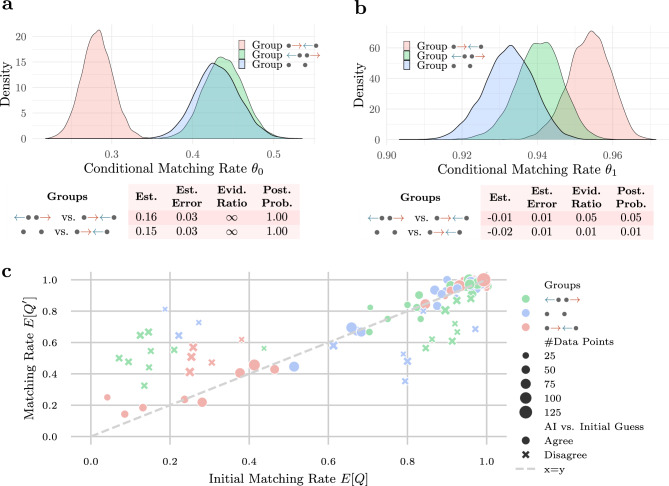
Results for groups ,  and . **a**) Results of Bayes A/B test when initial match $Q =0$ for groups ,  and : The plot shows the Bayes posterior of conditional matching rate $\theta _0$ for all three groups. The table shows the estimated difference in $\theta _0$, the estimation error, the evidence ratio (Bayes factor) and the posterior probability for the hypothesis stating that $\theta _0$ is larger in Group  (or Group ) than in Group . **b**) Results of Bayes A/B test when initial match $Q =1$ for groups ,  and : The plot shows the Bayes posterior of conditional matching rate $\theta _1$ for all three groups. The table shows the estimated difference in $\theta _1$, the estimation error, the evidence ratio (Bayes factor) and the posterior probability for the hypothesis stating that $\theta _1$ is larger in Group  (or Group ) than in Group . **c**) Empirical matching rate of participants with the optimal guess stratified by initial matching rate and whether the initial guess agrees with the AI confidence.

Figure 4a,b show the results of the Bayesian A/B tests for the groups ,  and . We observe that the support of the Bayes posterior for $\theta _0$ in groups  and  obtains higher values than in Group . Both A/B tests also show decisive evidence to support the hypothesis that $\theta _0$ in Group  and in Group  is greater than in Group  (Evidence Ratio $>100$ for both tests), with the estimated difference in $\theta _0$ greater or equal to 0.15 in both tests. The tests do not support the hypothesis that $\theta _1$ in groups  or  is greater than in Group  (Evidence Ratio $<1$ for both tests). However, the estimated difference in $\theta _1$ was only $-0.01$ for Group  and $-0.02$ for Group . These findings suggest that, in groups with higher degree of alignment, the AI was more helpful in correcting the non-optimal guess made initially by the participants, while being slightly more harmful and misleading the participants when participants’ initial guess was optimal.

For a more fine-grained understanding of how the degree of alignment influences the matching rates, we stratified the empirical matching rate $\theta =\mathbb {E}[Q']$ by the initial matching rate $\mathbb {E}[Q]$ of the participants and whether the initial guess agrees with the AI confidence. The results are shown in Fig. 4c. We observe that the regions of disagreement between AI guess and initial guess, where the AI helped or misled the participants, are larger in quantity and size for the groups with higher degree of alignment than for Group . This suggests that the potential to learn a better guess is higher when the AI is more aligned with the participants. Although in Group and  participants were misled by the AI for some games, we hypothesize that, if they would play more games, participants could eventually learn to trust their own confidence more for these games and rely on the AI confidence more for the games where they perform worse.

**Fig. 5:**
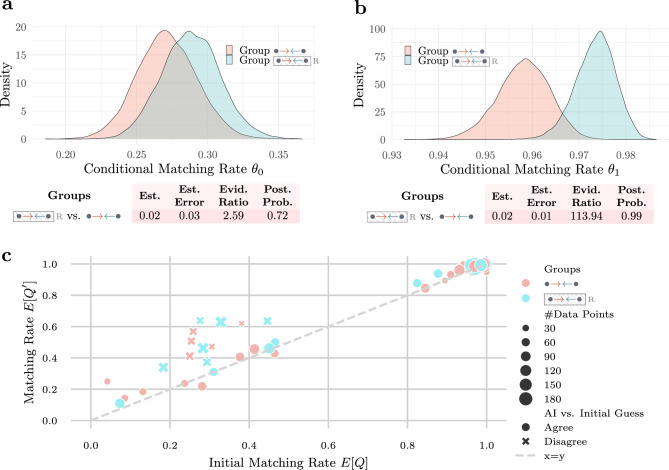
Results for groups  and . **a**) Results of Bayes A/B test when initial match $Q =0$ for groups  and : The plot shows the Bayes posterior of conditional matching rate $\theta _0$ for both groups. The table shows the estimated difference in $\theta _0$, the estimation error, the evidence ratio (Bayes factor) and the posterior probability for the hypothesis stating that $\theta _0$ is larger in Group  than in Group . **b**) Results of Bayes A/B test when initial match $Q =1$ for groups  and : The plot shows the Bayes posterior of conditional matching rate $\theta _1$ for both groups. The table shows the estimated difference in $\theta _1$, the estimation error, the evidence ratio (Bayes factor) and the posterior probability for the hypothesis stating that $\theta _1$ is larger in Group  than in Group . **c**) Empirical matching rate of participants with the optimal guess stratified by initial matching rate and whether the initial guess agrees with the AI confidence. Bins with 10 or less data points are omitted for sake of clarity.

Figure 5a,b show the results of the Bayesian A/B tests for the groups  and . We observe that the support of the Bayes posterior for $\theta _0$ and $\theta _1$ in Group  obtains higher values than in Group . While the A/B test only shows weak evidence to support the hypothesis that $\theta _0$ in Group  is greater than in Group  (Evidence Ratio 2.59), it shows decisive evidence to support the hypothesis that $\theta _1$ in Group  is greater than in Group  (Evidence Ratio $>100$). However, the estimated difference in both $\theta _0$ and $\theta _1$ was 0.02 showing only moderate improvement over Group . Nevertheless, these findings suggest that the realigned AI was both more helpful in correcting the non-optimal guess made by the participants and less harmful when participants’ initial guess was optimal. We hypothesize that this effect will be larger if the realignment of the AI decreases the alignment error further, for example, by using more fine grained bins during multicalibration or having more calibration data.

Figure 5c shows the empirical matching rate $\theta =\mathbb {E}[Q']$ stratified by the initial matching rate $\mathbb {E}[Q]$ of the participants and whether the initial guess agrees with the AI confidence. We also observe for Group  that the regions of disagreement between AI guess and initial guess are slightly more frequent than for Group . This suggests that the potential for participants to learn a better guess is greater with the realigned AI. In addition, we also observe that the multicalibration process does not create regions where the participants are misled by the AI when they were initially optimal unlike the regions present in groups  and .

## Discussion

Corvelo Benz and Gomez Rodriguez ^8^ stipulates that human-alignment is necessary for a rational human expert who places more (less) trust on predictions with higher (lower) AI confidence to be optimal. Their definition of rationality implies that on average an expert’s decisions are monotonically consistent in terms of their own and the AI’s confidence. Other types of decision consistency has been studied in psychology with two principal types being *linear consistency* ^15–17^ and *test-retest reliability* ^18,19^. Linear consistency—also known as response linearity, linear predictability, or cognitive control—concerns the extend to which a linear regression model estimated using underlying information available to an expert can reproduce the expert’s judgment (e.g., confidence) or decision ^19^. While test-retest reliability concerns the extend to which an expert makes identical decisions when exposed to the same information or stimuli in immediate successive occasions (internal consistency) and after longer periods of time (temporal stability) ^20,21^. In our study, we do not measure any kind of decision consistency for individual participants as the number of games played per participant is too small and each game played is of different game type by design. However, the results in Fig. 3 indicate monotonically consistent decisions on average across participants.

Classical economic theories of human decision making rely on the assumption that humans evaluate their decision options with the goal of maximizing the (expected) utility of their decision ^22,23^. However, in practice, human decisions have often been found to not achieve utility maximization due to the influence of various factors, such as cognitive biases or heuristics ^24,25^. In fact, we have also observed sub-optimality of the participants’ initial guess on average across all conditions in our study: The expected accuracy of the initial guess across participants in each group , , , and  is $67.3\%$, $68.1\%$, $66.2\%$, and $65.8\%$ while the expected accuracy of the optimal guess given the cards observed by the participants is $69.0\%$, $73.9\%$, $68.6\%$, and $68.5\%$, respectively (see Supplementary Information online for more details). This sub-optimality could be explained by various heuristics and biases described in psychology and behavioral economics research including *availability heuristics* ^26^, judging the likelihood of future events based on most recent past events, *representativeness* ^27^ or *base line neglect* ^28^, the use of a similar past event to judge the likelihood of a current event while ignoring current information, and *framing bias* ^29^, individuals making different decisions based on how identical information is presented. We aimed to minimize the effect of these cognitive biases on the results of the study by applying standard techniques such as randomizing the order of the different game types for each participant and using a single-blind study where instructions and exposition of the AI is identical across group conditions. Our results indicate that human-alignment is not only beneficial for monotonically consistent (rational) decision makers, but that even under the potential presence of cognitive biases (bounded rationality), the alignment of the AI to the human confidence can be beneficial to improve human decision making.

## Materials and methods

The study setup is available to play at https://hac-experiment.mpi-sws.org/?PROLIFIC_PID=test&STUDY_ID=test&SESSION_ID=test&LEVEL=B&GAME_BATCH=0. The study reported in this article was approved by the institutional review board of ETH Zurich under Institutional Review Board Protocol EK 2024-N-49 (“User Study on Human-Aligned Calibration for AI-Assisted Decision Making”) and we confirm that all experiments were performed in accordance with relevant guidelines and regulations according to the Declaration of Helsinki. All participants gave informed consent in advance.

### Recruitment of participants

We recruited in total 703 participants on Prolific for our study (average age, 38.44 years; age range, 18 to 79 years; gender, 346 male, 345 females, 8 non-binary, 4 not disclosed). Out of these participants, 302 participants were recruited to obtain calibration data for the multicalibration algorithm ^8^. For the different groups, balanced condition assignment and repeat-participant exclusion were performed directly on Prolific. Upon joining the study, each participant was assigned to Group  (100 participants), Group  (99 participants), Group  (102 participants), or Group  (100 participants). The study included an instruction block, a practice game, three comprehesion/attention checks, 24 game instances, and a set of end of game and end of study surveys, as well as a demographic survey. More information about the surveys, as well as an overview of the study timeline, is provided in Supplementary Fig. S5 to S12 online. The three attention checks were designed as simple game instances with a fraction of reds of $0\%,\ 75\%,$ and $100\%$ with no bias in the cards shown to the participants. The same three attention checks were used in each group, with the same configuration of cards shown to participants, and in the same order and place in the study timeline. We used the attention checks to filter out participants who performed out of the norm compared to most other participants: In more than one attention check, the confidence of the filtered participant was more than one standard deviation away from the mean confidence computed for each attention test over all groups. This resulted in on average 15.25 participants being excluded per group (15, 20, 17, and 9, respectively). We perform the same filtering procedure with participants recruited for obtaining calibration data (the mean and standard deviation being computed over only these participants), resulting in the exclusion of 42 participants.

### Game batches

For each group, we construct 20 game batches with one game pile (*r*, *a*, *z*) per type of game $(r,a)\in S$ by sampling *z* from the Wallenius’ non-central geometric distribution defined in Eq. 2. Similarly, we construct 60 game batches using the same distribution as in Group  to gather calibration data. Each participant completes one of the game batches consisting of 24 games such that, for each game batch, we obtain data from 4 to 6 participants. The 24 games are displayed to the participant in random order.

### Metrics and evaluation

In each game, participants are asked to state their initial confidence that the card picked is red in a range from 0 and 100. We transform this recorded confidence into a discretized confidence $h \in \mathcal {H}=\{\text {very low, low, high, very high}\}$ by dividing the confidence range into four regions of equal size ([0, 25], [26, 50], [50, 75], [76, 100]). When the initial confidence is 50, we assign the confidence value $\text {high}$ ($\text {low}$) if the initial guess is red (black).

To quantify the level of alignment error, we use the Maximum Alignment Error (MAE) and the Expected Alignment Error (EAE). The MAE is defined by Eq. 1 and the EAE is defined as

3 $$\begin{aligned} \text {EAE} = \frac{1}{N} \cdot \sum _{h \le h', a \le a'} [ P(Y = 1 \,\mid \,A=a, H=h) - P(Y = 1 \,\mid \,A=a', H=h') ]_{+}, \end{aligned}$$

where $$N=|\{h \le h', a \le a'\}|$$.

To run the Bayesian A/B tests, we use the “brms” package in R to fit the following model type

$$\begin{aligned} Q' \,\mid \,\text {trials}(Q') \sim 0 + g * Q + (1 \,\mid \,\text {participant}) \end{aligned}$$

using the binomial family. By default, this fits a logistic model for the proportions $\frac{\theta }{1-\theta }$. We set an uninformative standard normal prior $\mathcal {N}(0,1)$ for all parameters of the model. We fit two different models: one for groups , , and , and one for groups  and . The Bayesian leave-one-out cross-validation estimate of the expected log pointwise predictive probabilities (ELPD) for the former model is $-815.2$ (standard error 26.6, $\#$observations 497) and for the latter model is $-547.7$ (standard error 23.4, $\#$observations 352). For comparison, a null model with uniform distribution across 25 possible outcomes (0-24) would obtain an ELPD of $\log (1/25)=-3.22$ per observation, so our models—with ELPD of $-1.64$ and $-1.55$ per observation—perform better than uniform random guessing. Given the fitted models, we use the hypothesis function of “brms” to run the following Bayesian A/B tests: , , , ,  and  where the second subscript indicates the group condition. In the supplementary information online, we have also performed a frequentist analysis using the Boschloo’s exact test (independently) on the same set of alternative hypotheses. The results are summarized in Supplementary Table S2 to S4 online. The Boschloo’s exact tests were conducted using the SciPy library in Python.

### Realignment algorithm

Since participants’ confidence partitions the game instances into disjoint subspaces, we realign the AI model using the multicalibration algorithm based on uniform mass binning outlined by Corvelo Benz and Gomez Rodriguez ^8^. The algorithm partitions each subspace into $N=5$ uniform mass bins based on the AI confidence. Then, it computes the empirical mean fraction of reds for those game instances in the same bin. This empirical mean is computed using the true fraction of reds of each game instance as it gives more reliable estimates than using Bernoulli samples for small sample size. Finally, the algorithm returns this empirical estimate as the re-aligned AI confidence for new game instances in the same subspace—with the same participant’s confidence—falling into the same AI confidence bin. Since the AI confidence obtains discrete levels in our study, we add a small random uniform noise (in $[0,\nicefrac {1e-10}{1+1e-10}]$) to the AI confidence, both during training and deployment for Group , for the theoretical guarantees of the algorithm to hold.

## Supplementary Information

Supplementary Information.

## Data Availability

The datasets generated and/or analyzed during the current study are available in the Human-Alignment-Study repository https://github.com/Networks-Learning/human-alignment-study/releases/tag/v1.1
