# Supplementary material for: Human-alignment influences the utility of AI-assisted decision making
Source: Sci Rep. 2025 Aug 9;15:29154. doi: 10.1038/s41598-025-12205-1 (PMC12335573; doi:10.1038/s41598-025-12205-1)
Supplement: Supplementary file 1 — Supplementary Information. [file 41598_2025_12205_MOESM1_ESM.pdf]

Human-Alignment Influences the Utility of AI-assisted Decision Making

Nina L. Corvelo Benz[1,2,*] and Manuel Gomez Rodriguez[1]

[1]Max Planck Institute for Software Systems, {ninacobe, manuelgr}@mpi-sws.org
[2]Department of Biosystems Science and Engineering, ETH Zurich

Supplementary Information

1 Additional Experimental Results

We make two observations matching the theoretical assumptions and results in Corvelo Benz and Gomez Rodriguez [1] in the study.

Supplementary Fig. S1 shows the probabilities of guessing red given the initial and final guess of participants. We observe that participants' initial guess is monotonic in their initial confidence, and that the final guess is monotonic in their initial confidence and the AI confidence.

Given the record of initial confidence $h_{i,j}$, the initial $d_{i,j}$ and final $d'_{i,j}$ color guess made by the participant j in the game instance i, we compute the best monotone π_{mono} and best joint π_{joint} guess for all games played. The best joint guess maximizes the expected accuracy of the guess given only confidence h_i and a_i—the guess is red (black) if the mean probability of a red card for game instances with confidence h_i and a_i is higher (lower) than 50%. The best monotone guess maximizes the expected accuracy among guesses made by rational decision makers—guesses that are monotone in confidence h_i and a_i.

We measure the expected accuracy of each guess averaged across participants and games,

$$\sum_j \frac{1}{\#\text{participants}} \cdot \sum_i \frac{1}{24} \cdot \mathbb{E}_{C_i \sim Be(r_i)}[\mathbb{1}[\pi(i,j) = C_i]] \tag{1}$$

where $\pi(i,j) \in \{d_{i,j}, d'_{i,j}, \pi_{\text{joint}}(i,j), \pi_{\text{mono}}(i,j)\}$ denotes the guess evaluated. For each guess, Supplementary Fig. S1 shows a heatmap of the decision probabilities of guessing red stratified by participants' initial confidence and AI confidence shown, and averaged over participants. Supplementary Fig. S2 shows the utility in terms of expected accuracy (Eq. 1) of the best joint guess and the best monotone guess that could be made by the participants based on their own and the AI confidence values. Although the expected accuracy of the best joint guess is mostly equivalent across groups and the expected accuracy of the AI alone is the same by design, the expected accuracy of the best monotone guess is lower in groups with lower degree of alignment. However, while the difference in the best monotone guess is barely noteable, the difference in the expected accuracy of the final guess is more prominent.

1.1 Expected Calibration Error

1.2 Frequentist Analysis of Study Data

To complement the Bayesian A/B tests performed in the main manuscript, we perform the Boschloo's exact test independently for the following alternative hypotheses for the conditional matching rates θ_0 and θ_1: (i) $\theta_{0,\leftarrow\bullet\bullet\rightarrow} > \theta_{0,\bullet\rightarrow\leftarrow\bullet}$, (ii) $\theta_{1,\leftarrow\bullet\bullet\rightarrow} > \theta_{1,\bullet\rightarrow\leftarrow\bullet}$, (iii) $\theta_{0,\bullet\quad\bullet} > \theta_{0,\bullet\rightarrow\leftarrow\bullet}$, (iv) $\theta_{1,\bullet\quad\bullet} > \theta_{1,\bullet\rightarrow\leftarrow\bullet}$, (v)

Table S1: Expected Calibration Error (ECE) measured empirically for each group. As expected, all values are relatively low. The AI in group ●→←● , while empirically the least aligned, also obtains lower ECE than in groups ←●●→ and ● ● . The post-processing algorithm does not significantly reduce ECE of the AI in group [●→←●] R .

Group	ECE
←●●→	0.090
● ●	0.055
●→←●	0.036
[●→←●] R	0.033

$\theta_{0,\boxed{\bullet\rightarrow\leftarrow\bullet}\mathrm{R}} > \theta_{0,\bullet\rightarrow\leftarrow\bullet}$, and (vi) $\theta_{1,\boxed{\bullet\rightarrow\leftarrow\bullet}\mathrm{R}} > \theta_{1,\bullet\rightarrow\leftarrow\bullet}$, where the second subscript indicates the group condition. Tables S2 and S3 show the corresponding contingency tables used in the test and Table S4 summarizes the results, which support the conclusions derived from the Bayesian analysis. For a significance level of 5%, we have evidence to reject the corresponding null hypothesis in favor of the alternative hypotheses $\theta_{0,\leftarrow\bullet\bullet\rightarrow} > \theta_{0,\bullet\rightarrow\leftarrow\bullet}$, $\theta_{0,\bullet\ \bullet} > \theta_{0,\bullet\rightarrow\leftarrow\bullet}$, and $\theta_{1,\boxed{\bullet\rightarrow\leftarrow\bullet}\mathrm{R}} > \theta_{1,\bullet\rightarrow\leftarrow\bullet}$ while we found no evidence in the other tests. Table S4 summarizes the results.

Table S2: Contingency table for Boschloo's exact tests when initial match $Q = 0$.

	←●●→	● ●	●→←●	[●→←●] R
**Final guess optimal** ($Q' = 1$)	199	153	164	196
**Final guess not optimal** ($Q' = 0$)	254	202	437	467

Table S3: Contingency table for Boschloo's exact tests when initial match $Q = 1$.

	←●●→	● ●	●→←●	[●→←●] R
**Final guess optimal** ($Q' = 1$)	1490	1434	1377	1477
**Final guess not optimal** ($Q' = 0$)	97	107	62	44

Table S4: Results of Boschloo's exact tests summarized.

**Initial Match Q=0**			**Initial Match Q=1**		
**Alt. Hypth.**	**p-value**	**p<0.05**	**Alt. Hypth.**	**p-value**	**p<0.05**
$\theta_{0,\leftarrow\bullet\bullet\rightarrow} > \theta_{0,\bullet\rightarrow\leftarrow\bullet}$	$9.98e-09$	yes	$\theta_{1,\leftarrow\bullet\bullet\rightarrow} > \theta_{1,\bullet\rightarrow\leftarrow\bullet}$	0.989	no
$\theta_{0,\bullet\ \bullet} > \theta_{0,\bullet\rightarrow\leftarrow\bullet}$	$3.24e-07$	yes	$\theta_{1,\bullet\ \bullet} > \theta_{1,\bullet\rightarrow\leftarrow\bullet}$	0.993	no
$\theta_{0,\boxed{\bullet\rightarrow\leftarrow\bullet}\mathrm{R}} > \theta_{0,\bullet\rightarrow\leftarrow\bullet}$	0.187	no	$\theta_{1,\boxed{\bullet\rightarrow\leftarrow\bullet}\mathrm{R}} > \theta_{1,\bullet\rightarrow\leftarrow\bullet}$	0.023	yes

References

[1] Nina Corvelo Benz and Manuel Rodriguez. Human-aligned calibration for ai-assisted decision making. *Advances in Neural Information Processing Systems*, 36, 2024.

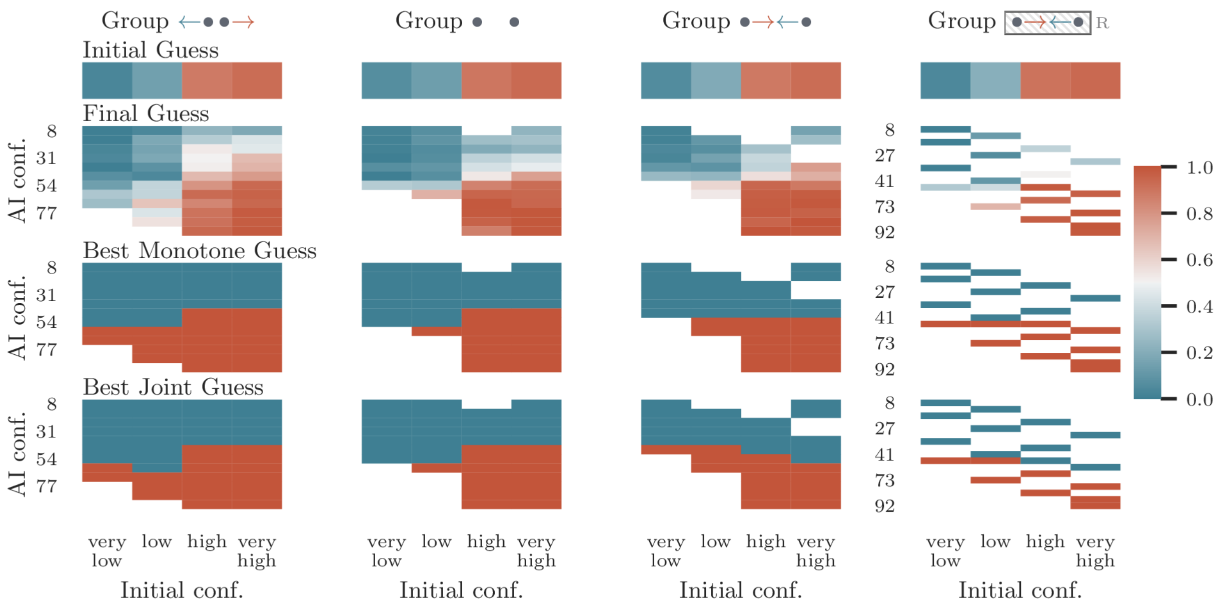

Figure S1: Heatmap of decision probabilities of guessing red stratified by participants' initial confidence and AI confidence shown, and averaged over participants. The initial confidence recorded is discretized into four bins—very low, low, high, very high—denoting the confidence of the participants that the color of the picked card will be red. Bins with 10 or less data points are not displayed.

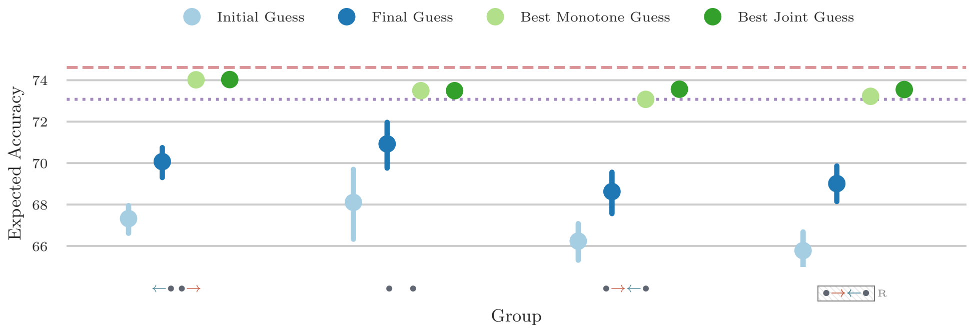

(a) Expected accuracy averaged across participants and games for each group and type of guess. Red dashed line indicates the expected accuracy of the optimal guess π^* and purple dotted line indicates the expected accuracy of the AI playing alone. Both are by design of the study equal across groups and, thus, just drawn as lines.

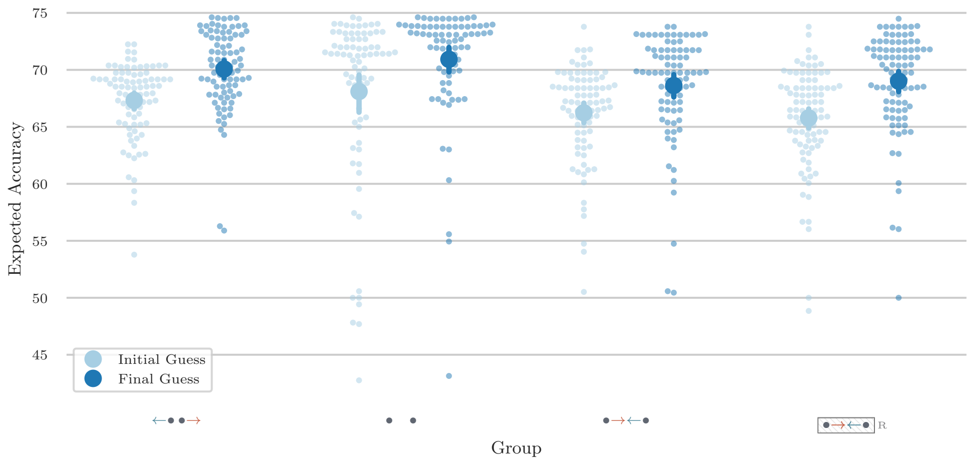

(b) Expected accuracy of each individual participant averaged across games for each group for the initial and final guess.

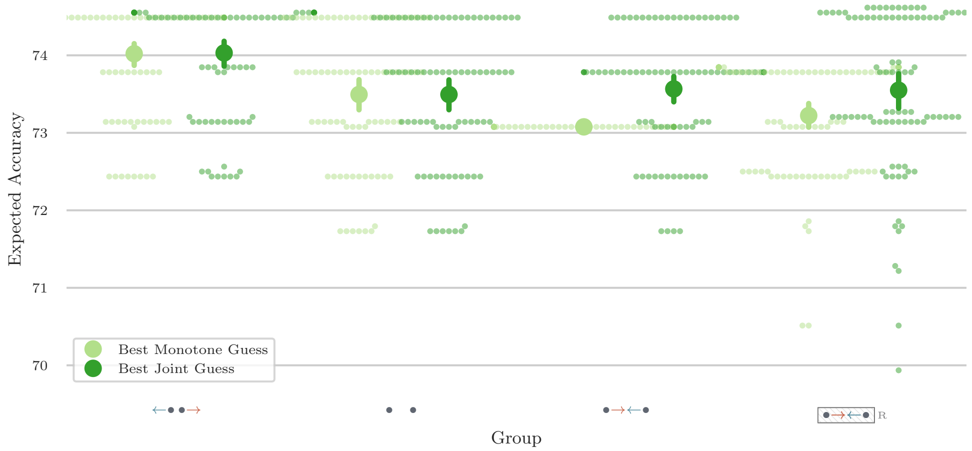

(c) Expected accuracy of each individual participant averaged across games for each group for the best joint and best monotone guess. Some points were not drawn due to space constraints.

Figure S2: Accuracy across groups and guesses in expectation. Plot (a) compares means and 95% confidence intervals, while plot (b) and (c) show individual expected accuracies of each participant as well.

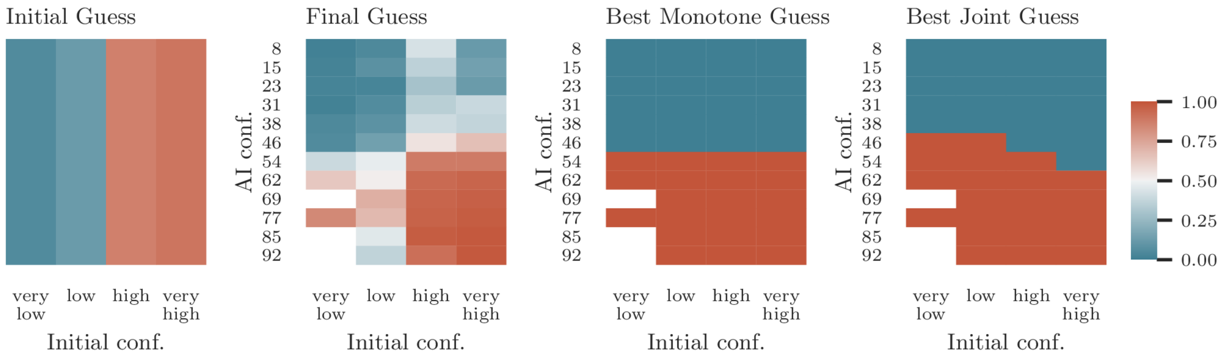

(a) Heatmap of decision probabilities of guessing red stratified by participants' initial confidence and AI confidence shown, and averaged over participants. The initial confidence recorded is discretized into four bins—very low, low, high, very high—denoting the confidence of the participants that the color of the picked card will be red. Bins with 10 or less data points are not displayed.

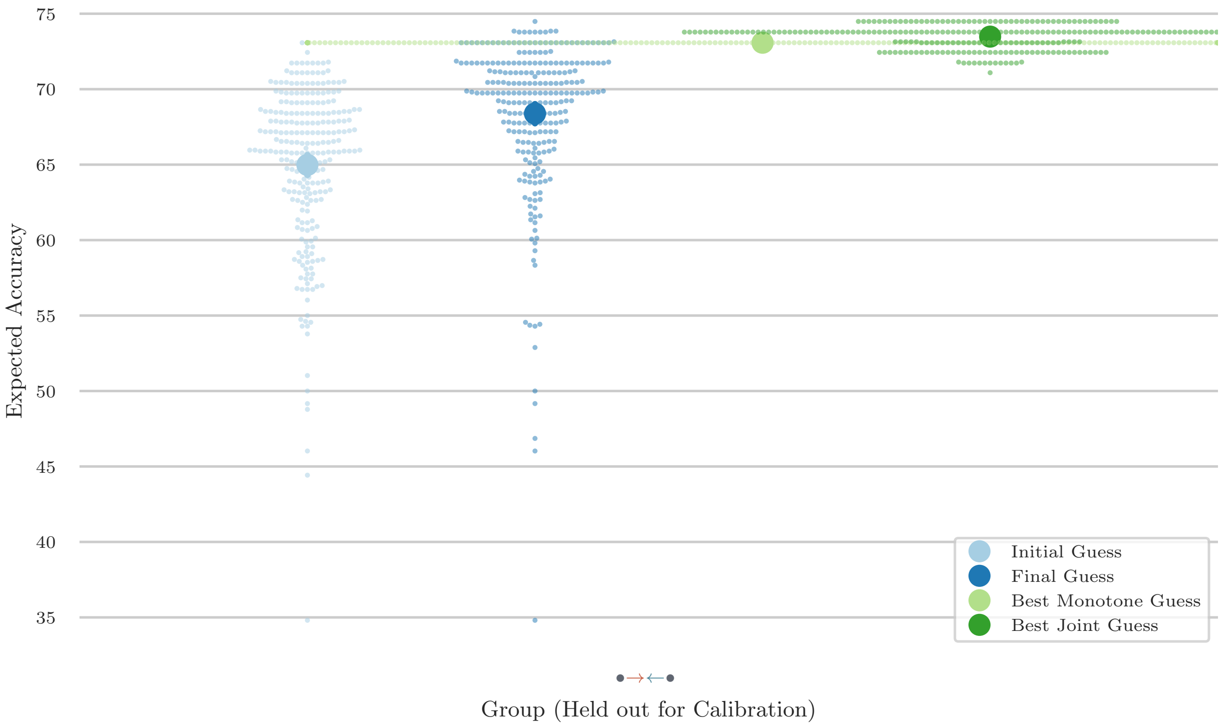

(b) Expected accuracy of each individual participant averaged across games for each guess type. Means over participants are plotted as large circle.

Figure S3: Summarization plots for held-out calibration data from group ●→←● . Plot (a) shows a heatmap of the probability of guessing red. Plot (b) shows a swarm plot of the expected accuracy of each individual participant averaged across games for each guess type.

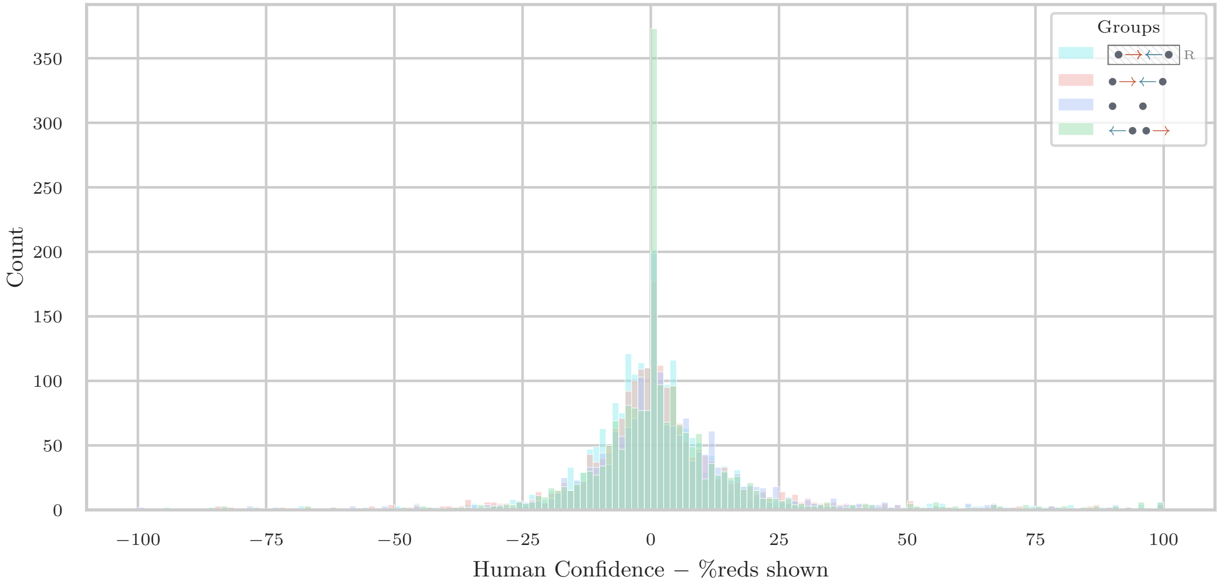

Figure S4: Histogram of the difference between reported initial confidence and percentage of reds shown to participants for each group. We observe no difference in distribution across groups.

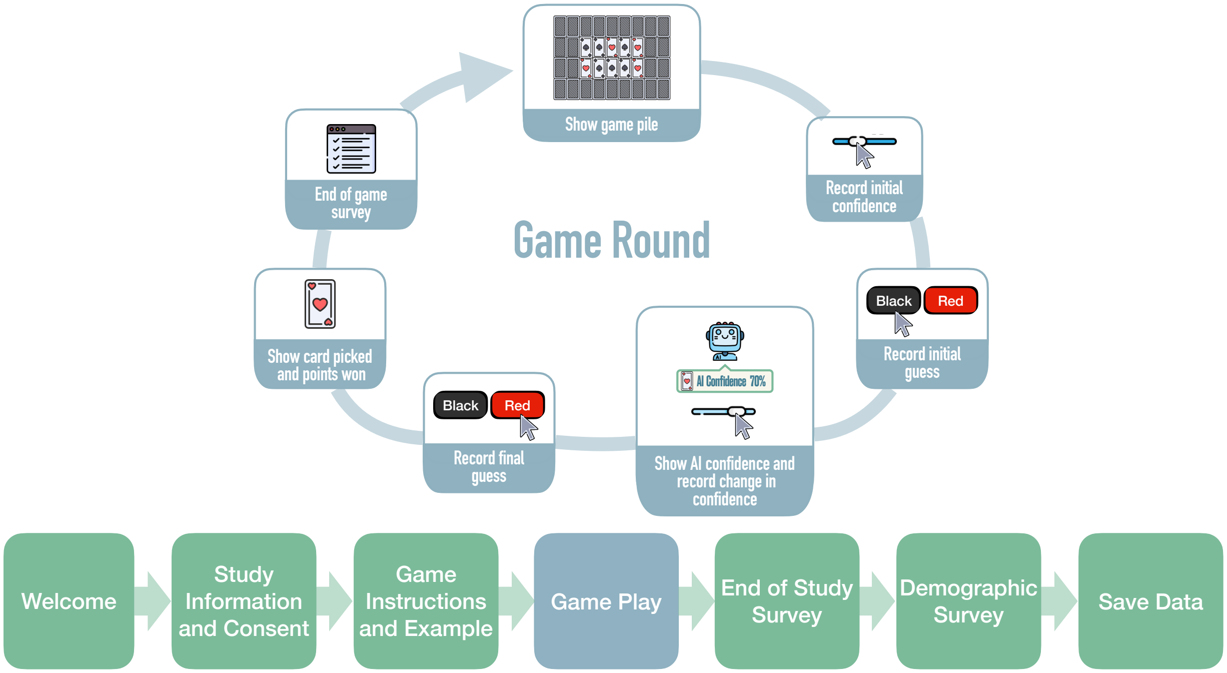

Figure S5: Flow chart showing an overview of the study timeline. Screenshots of an example game round can be found in Supplementary Fig. S6 to S9. A screenshot of the end of game survey and end of study survey can be seen in Supplementary Fig. S10. The full study setup can be found at `https://hac-experiment.mpi-sws.org/?PROLIFIC_PID=test&STUDY_ID=test&SESSION_ID=test&LEVEL=B&GAME_BATCH=0`.

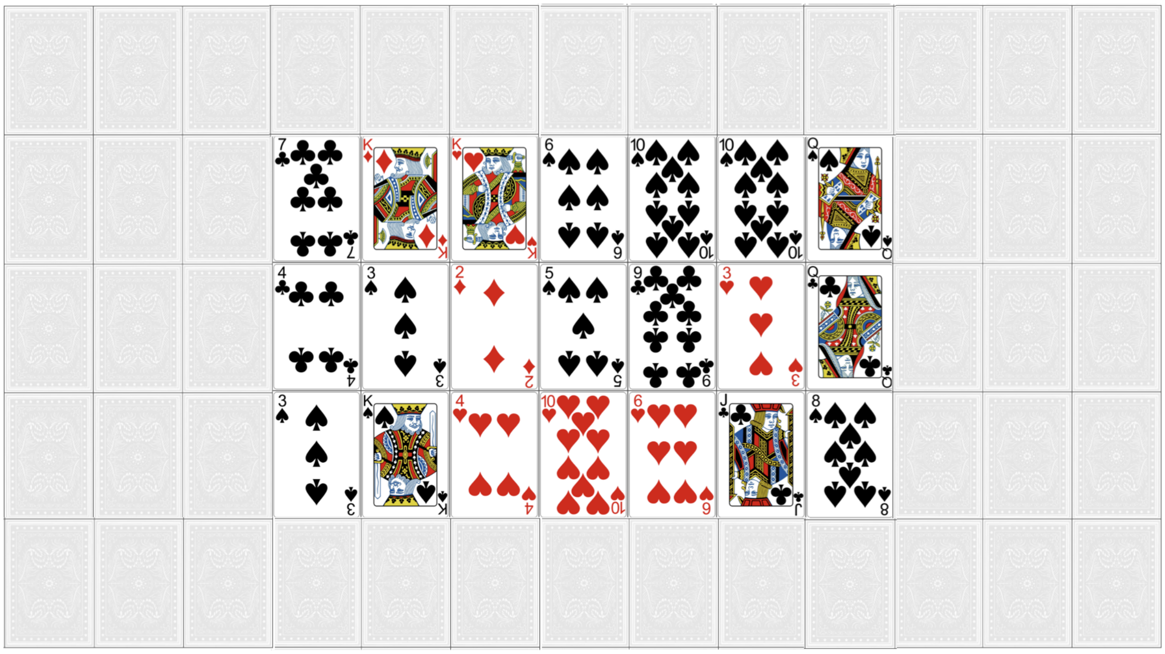

Figure S6: Example of a game pile shown to participants.

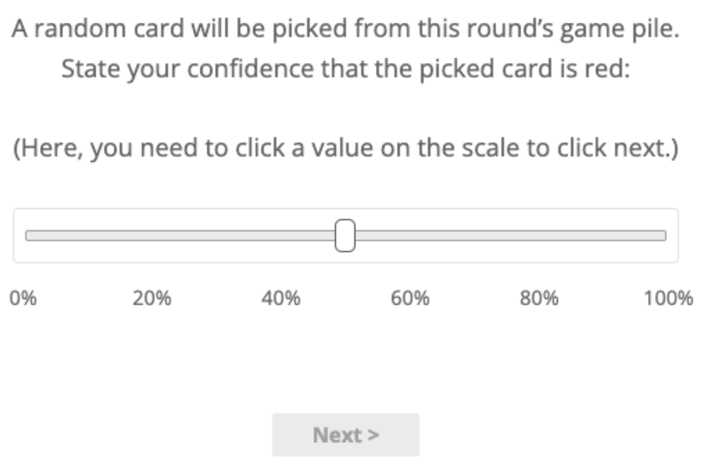

(a) Record initial confidence

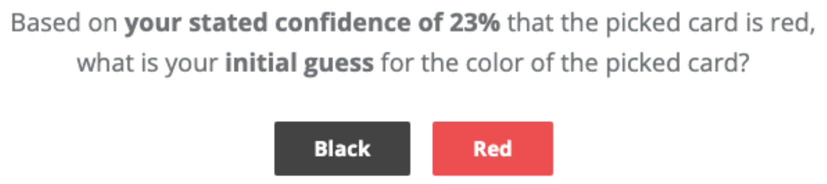

(b) Record initial guess

Figure S7: Screenshots of game round steps where participants are asked for their initial confidence and guess before being shown the AI confidence.

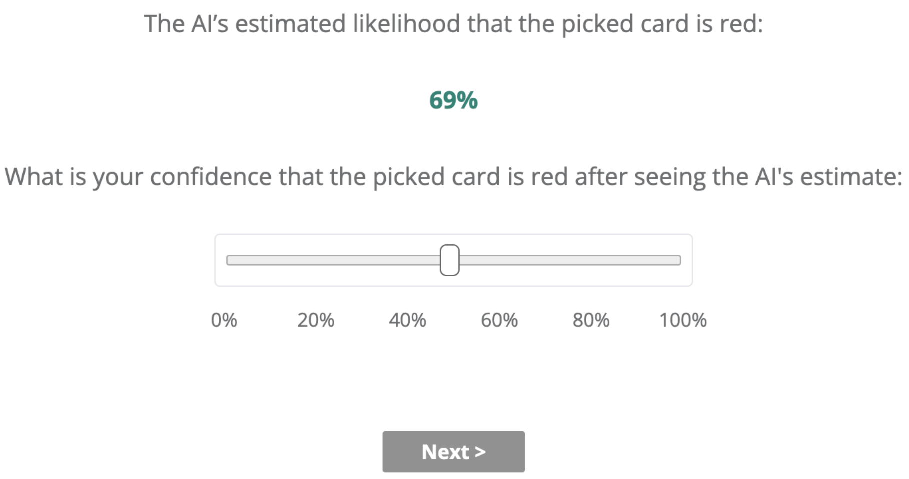

(a) Show AI confidence and record change in confidence of the participant

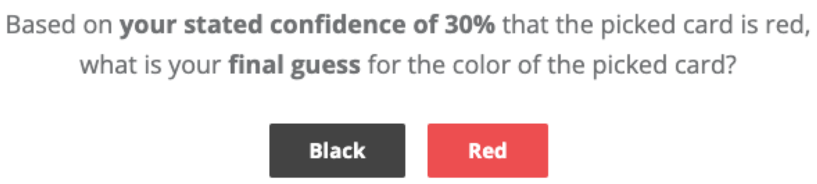

(b) Record final guess

Figure S8: Screenshots of game round steps where participants are shown the AI confidence and asked for their confidence and final guess.

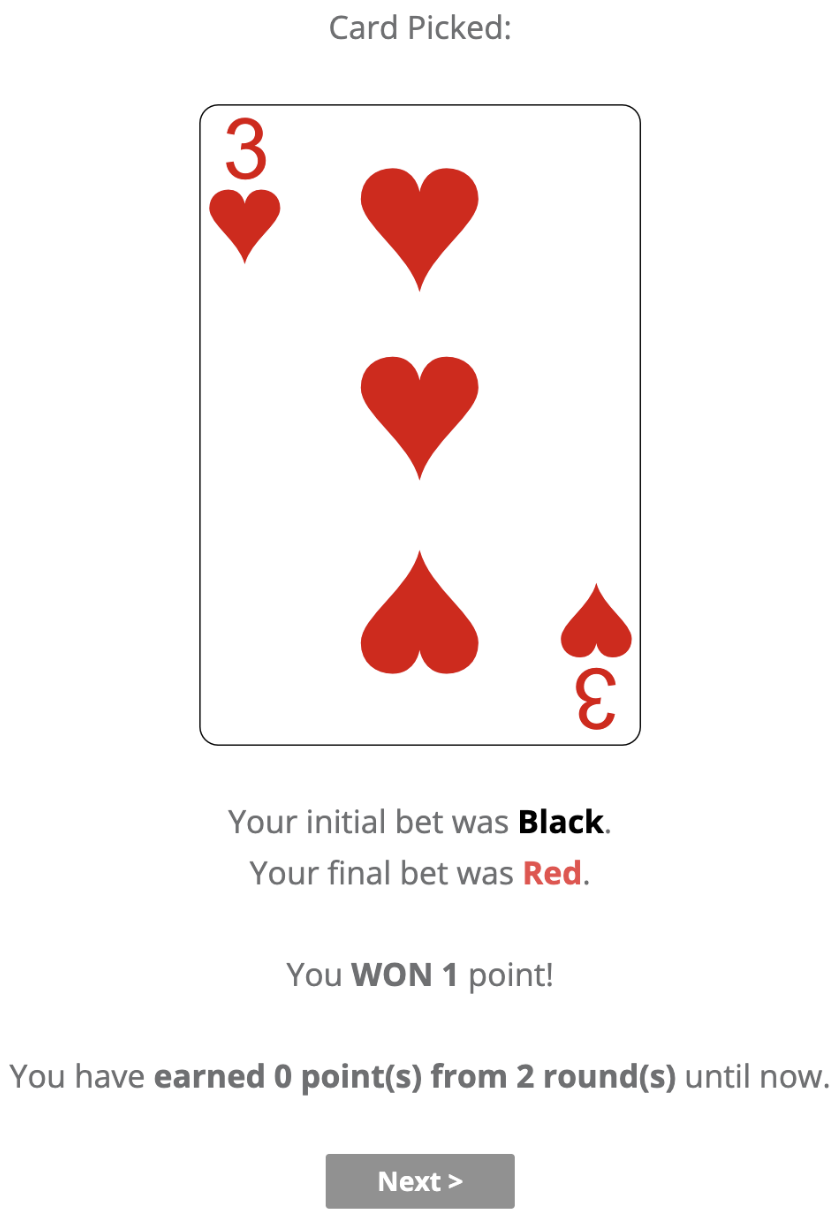

Figure S9: Screenshot of how participants are shown the card picked and points won.

State how much you agree with following statement about the AI.

	Strongly Disagree	Disagree	Neutral	Agree	Strongly Agree
I think the AI's estimate was accurate in this round.	◯	◯	◯	◯	◯

Choose the statement that best applies to this round. *

◯ I won points in this round because I changed my guess based on the AI's estimate.

◯ I lost points in this round because I changed my guess based on the AI's estimate.

◯ The AI's estimate did not change my guess in this round.

Next Round >

(a) End of game survey

Please answer the following questions about your overall thoughts on the AI during the game.

State how much you agree with following statements about the AI.

	Strongly Disagree	Disagree	Neutral	Agree	Strongly Agree
Overall, I think the AI's estimates were accurate.	◯	◯	◯	◯	◯
Overall, the AI's estimates affected my confidence.	◯	◯	◯	◯	◯
Overall, I trust the estimates of the AI.	◯	◯	◯	◯	◯

Choose the statement that best applies. *

◯ Overall, the AI's estimates changed my guess for the better, and I would have gained less points without the AI.

◯ Overall, the AI's estimates changed my guess for the worse, and I would have gained more points without the AI.

◯ Overall, the AI's estimates did not change my guess, and I would have gained a similar amount of points without the AI.

◯ Overall, the AI's estimates did not change my guess, and I believe I would have gained less points by trusting the AI.

◯ Overall, the AI's estimates did not change my guess, but I believe I would have gained more points by trusting the AI.

Next >

(b) End of study survey

Figure S10: Screenshot of the end of game and end of study surveys filled out by participants.

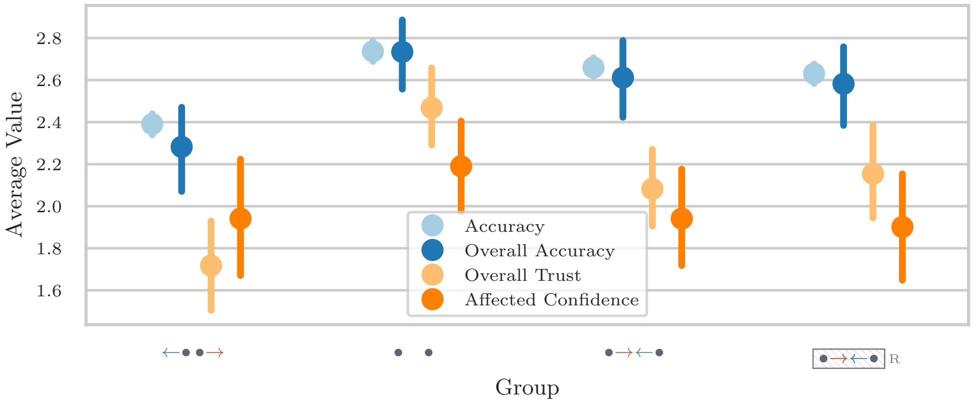

(a) Averaged value of the likert scale—strongly disagree (0) to strongly agree (5)—in the end of game and end of study survey for following statements: "I think the AI's estimate was accurate in this round." (Accuracy), "Overall, I think the AI's estimates were accurate." (Overall Accuracy), "Overall, the AI's estimates affected my confidence." (Affected Confidence) and "Overall, I trust the estimates of the AI." (Overall Trust).

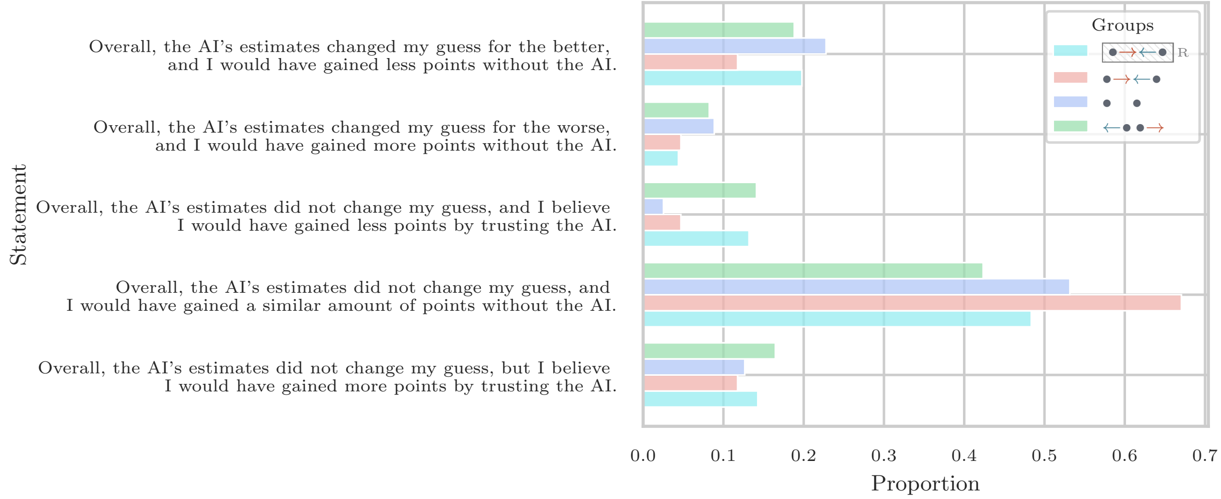

(b) Frequency of statement selected by participants to best apply to their whole game experience at the end of study survey.

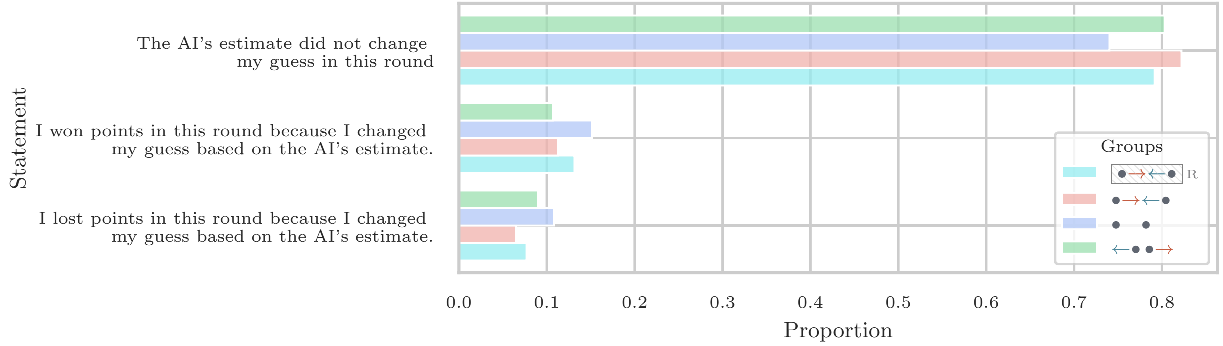

(c) Frequency of statement selected by participants to best apply to their game experience at the end of game survey.

Figure S11: Summarized responses to end of game and end of study surveys.

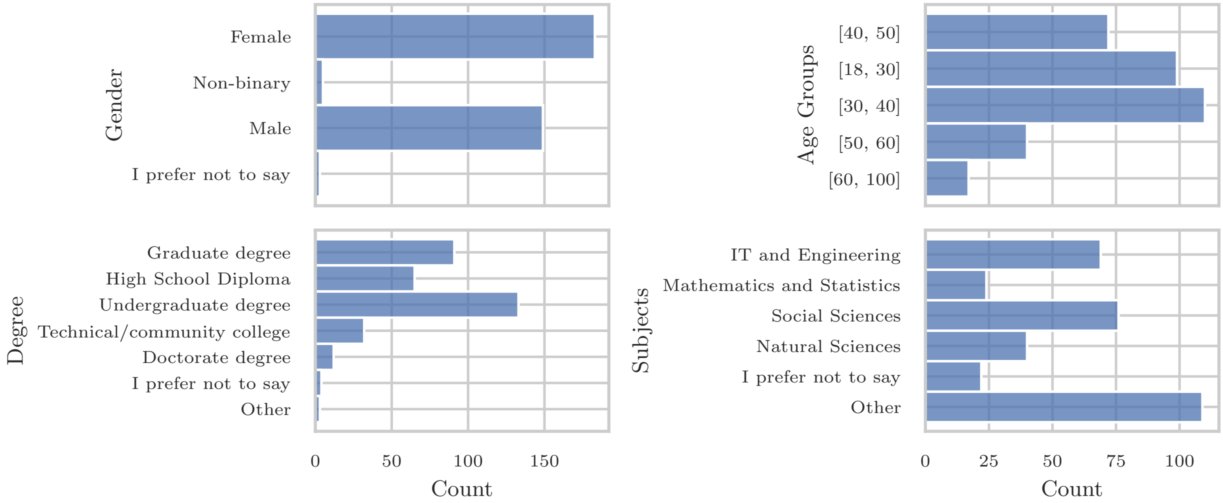

Figure S12: Demographic information provided by participants including gender, age, highest obtained degree and subject of degree.

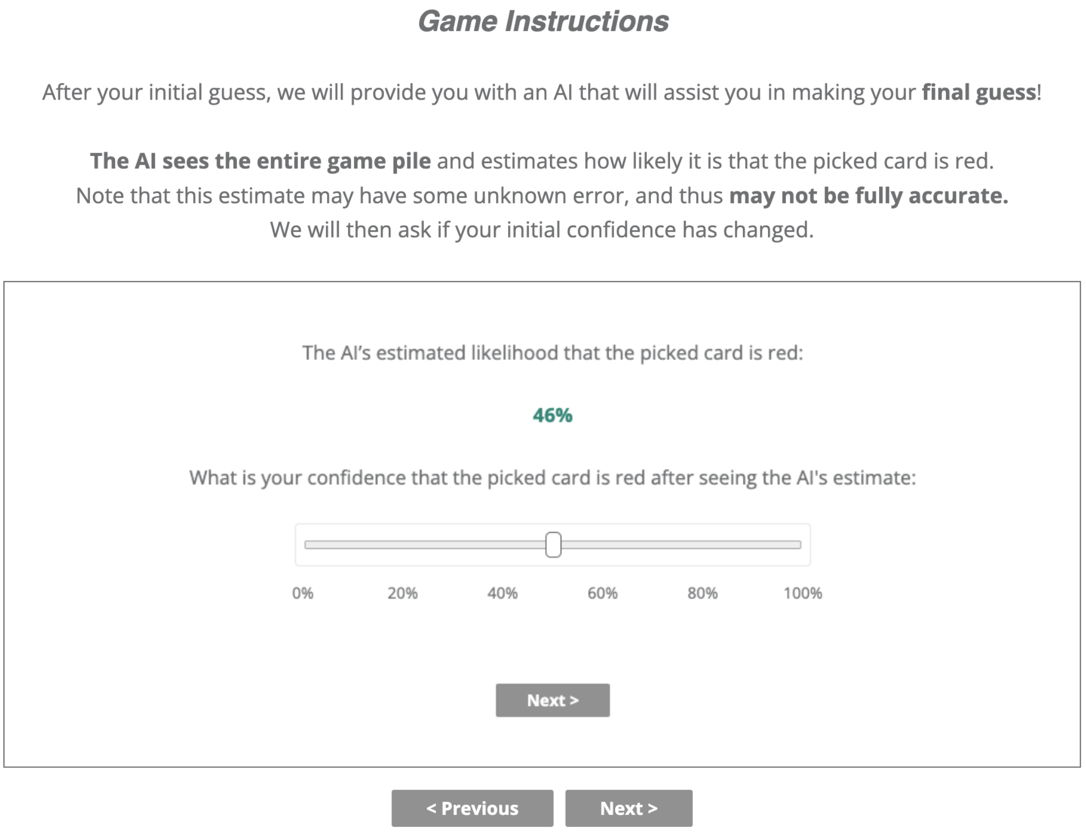

Figure S13: Introduction of the AI shown to the participants during game instructions.
